# COVID-19 Testing and Diagnostics: A Review of Commercialized Technologies for Cost, Convenience and Quality of Tests

**DOI:** 10.3390/s21196581

**Published:** 2021-10-01

**Authors:** Ashler Benda, Lukas Zerajic, Ankita Ankita, Erin Cleary, Yunsoo Park, Santosh Pandey

**Affiliations:** Department of Electrical and Computer Engineering, Iowa State University, Ames, IA 50011, USA; ashlerb@iastate.edu (A.B.); lzerajic@iastate.edu (L.Z.); ankita@iastate.edu (A.A.); eecleary@iastate.edu (E.C.); yunsoopk@iastate.edu (Y.P.)

**Keywords:** COVID-19 diagnostic test, SARS-CoV-2 detection, at home test, rapid antigen test, serology test, mass testing, FDA authorized test kit, universal access

## Abstract

Population-scale and rapid testing for SARS-CoV-2 continues to be a priority for several parts of the world. We revisit the in vitro technology platforms for COVID-19 testing and diagnostics—molecular tests and rapid antigen tests, serology or antibody tests, and tests for the management of COVID-19 patients. Within each category of tests, we review the commercialized testing platforms, their analyzing systems, specimen collection protocols, testing methodologies, supply chain logistics, and related attributes. Our discussion is essentially focused on test products that have been granted emergency use authorization by the FDA to detect and diagnose COVID-19 infections. Different strategies for scaled-up and faster screening are covered here, such as pooled testing, screening programs, and surveillance testing. The near-term challenges lie in detecting subtle infectivity profiles, mapping the transmission dynamics of new variants, lowering the cost for testing, training a large healthcare workforce, and providing test kits for the masses. Through this review, we try to understand the feasibility of universal access to COVID-19 testing and diagnostics in the near future while being cognizant of the implicit tradeoffs during the development and distribution cycles of new testing platforms.

## 1. Introduction

To limit the spread of SARS-CoV-2, aggressive and scalable deployment of COVID-19 testing resources has been a priority of health and administrative officials worldwide. A COVID-19 diagnostic test is advisable for individuals experiencing COVID-19 symptoms or those exposed to persons with suspected or confirmed COVID-19 illness [1,2]. The COVID-19 test is also advised for travel purposes, recreation, social gatherings and professional meetings, or can be enforced at the workplace by employers [3]. Patients with a confirmed COVID-19 infection develop fever and/or acute respiratory illness which may lead to death [2]. Timely test results help provide informed recommendations to the patient, thereby protecting the front-line workers and limiting the COVID-19 transmission to others in close contact [4,5].

Early investments in new diagnostic technologies with rapid and decentralized testing have been vital in minimizing the negative health and socioeconomic impacts of SARS-CoV-2 [6,7,8]. In April 2020, the U.S. National Institute of Health (NIH) launched the Rapid Acceleration of Diagnostics (RADx) Initiative to ramp up development, commercialization, and implementation of COVID-19 testing technologies [9]. The goal of the RADx Initiative was to develop innovative diagnostic tests that are fast, accurate, easy-to-use, and easily accessible at home and point of care, particularly to population groups that are vulnerable and most impacted by COVID-19 [9]. On a parallel front, the Foundation for Innovative New Diagnostics (FIND), a Geneva-based not-for-profit organization, has led international partnerships for scaled-up development and delivery of COVID-19 tests through its Access to COVID-19 Tools (ACT) Accelerator [8]. Despite these commendable efforts, the pandemic continues to rattle several parts of the world, especially the low- and middle-income households where testing sites are inaccessible and test kits are cost-prohibitive or in limited supply [10]. For the long-term containment of the virus, the current goal of governments and institutions worldwide lies in the equitable distribution of COVID-19 testing resources in a sustainable manner [7,10].

Our objective here is to review the commercialized in vitro diagnostic tests for the detection of SARS-CoV-2, primarily focusing on tests granted Emergency Use Authorization (EUA) by the U.S. Food and Drug Administration (FDA). We expand upon previous review papers on COVID-19 test assays published since the start of the global pandemic [7,10,11,12] by providing a comprehensive review of current testing methods available from the companies granted FDA EUA. Various attributes of COVID-19 testing platforms are tabulated and comparative studies on the evaluation and validation of available tests are discussed. We try to understand the implicit tradeoffs of cost, convenience, and quality of available COVID-19 tests based on the information gathered from websites, databases, and blogs of product companies, federal and medical agencies, and governing bodies. The outline of this paper is as follows. Section 2 provides a brief overview of in vitro diagnostic (IVD) tests for SARS-CoV-2, and the different at-home and population-scale testing strategies. Section 3 discusses the standardized regulatory and reporting guidelines for device manufacturers and testing facilities. Section 4 and Section 5 describe the molecular diagnostic tests for SARS-CoV-2 using respiratory specimens and saliva specimens, respectively. Section 6 describes the rapid antigen tests for SARS-CoV-2, including lateral flow assays with visual readout. Section 7 talks about the deployment of COVID-19 test kit vending machines and Testing-as-a-Service platforms. Section 8 deals with serology or antibody tests for SARS-CoV-2, such as assays to measure immunoglobulins or total antibodies in blood. Section 9 describes the diagnostic tests for the management of COVID-19 patients that track the biomarkers of inflammation (e.g., interleukin-6). Section 10 discusses the challenges and outlook related to lowering the testing costs, improving the performance metrics, building a robust supply chain, and better containment of new variants (such as the Delta and Lambda Variants of the Coronavirus).

## 2. Diagnostic Tests for SARS-CoV-2: An Overview

**Figure 1:**
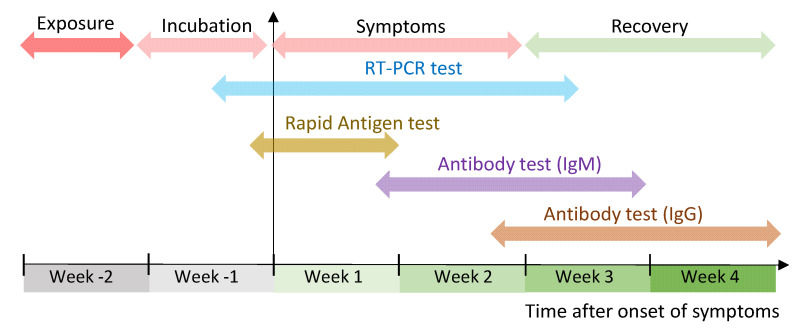
Suitability of different types of in vitro diagnostic tests during the progression of COVID-19 infection. Approximate time intervals of SARS-CoV-2 exposure, incubation, symptoms, and recovery are illustrated. The detectability of the viral genetic material, viral proteins, and antibodies (IgM and IgG) to the virus are shown for the RT-PCR test, rapid antigen test, and antibody tests, respectively.

The FDA has granted Emergency Use Authorization (EUA) to several in vitro diagnostic tests for SARS-CoV-2 [6]. These IVD tests are grouped into three categories − diagnostic tests (i.e., molecular tests and ‘rapid’ antigen tests), serology or antibody tests, and tests for the management of COVID-19 patients depending on their suitability to detect the disease parameters from the onset of symptoms (Figure 1) [10,11,13]. The first type of IVD tests are diagnostic tests that detect parts of the SARS-CoV-2 virus for the diagnosis of active COVID-19 infection (i.e., where there is active shedding of the virus). The samples for diagnostic tests are collected using a nasal or throat swab, or from saliva by spitting into a tube. Sometimes, secretions from the lower respiratory tract, such as sputum and bronchoalveolar lavage fluid, are also used for confirmatory tests [7]. The second type of IVD tests are serology or antibody tests that measure the presence of antibodies to SARS-CoV-2 from serum, plasma or whole blood from finger stick or veins. The detected antibodies are indicative of previous exposure to the virus or immune status of the individual. The third type of IVD tests evaluate the severe inflammatory response in patients with confirmed COVID-19 infection, typically by measuring the levels of interleukin-6 (IL-6) in serum, plasma or whole blood [11].

The FDA and Centers for Disease Control and Prevention (CDC) have taken several steps to speed up scientific discovery towards commercialization and reduce the burden of test validation for new test developers [6]. For instance, the FDA allows any new developer to leverage the data from previous EUA-authorized assays by submitting a right of reference from the sponsor. By doing so, new developers can leverage the CDC’s in silico and cross reactivity tests, rather than repeating the tests on their assays. As an example, the CDC has granted the right of reference for its CDC 2019-nCoV Real-Time RT-PCR Diagnostic Panel and Influenza SARS-CoV-2 (Flu SC2) Multiplex Assay to any entity seeking an FDA EUA for a COVID-19 diagnostic device or developing a multi-analyte respiratory panel including SARS-CoV-2 [6].

The FDA’s In Vitro Diagnostics EUAs website provides a list of companies whose COVID-19 test kits have received Emergency Use Authorization (EUA) [6]. At the time of this publication, there were roughly 243 entries for authorized SARS-CoV-2 molecular diagnostic tests, 79 entries for authorized SARS-CoV-2 serology or antibody tests, and 3 entries for authorized in vitro diagnostic tests for the management of COVID-19 patients. In this review, we primarily focus on these COVID-19 test kits granted FDA EUA. On a cautionary note, IVD tests are not suitable for COVID-19 patients showing emergency warning signs, such as “breathing difficulty, persistent pain or pressure in the chest, inability to wake up or stay awake, new confusion, and bluish lips or face” [3].

Within the United States, IVD tests are offered at local health centers and select pharmacies such as Walgreens, CVS Health, Rite Aid, Walmart, Health Mart, eTrueNorth, and Topco. The U.S. Families First Coronavirus Response Act ensures that COVID-19 tests are freely available to anyone. Some at-home IVD tests produce results within a few minutes, while others could require the patient to mail the sample to a testing lab for analysis. Apart from at-home testing, laboratories and testing sites are pursuing alternate testing strategies for faster screening of COVID-19 infections, such as pooled testing, screening programs, and surveillance testing [7,10]. Pooled testing is performed where the samples from different individuals are combined into one sample and tested together [5]. It is suitable for testing multiple people for COVID-19 in a shorter time, particularly in low-resource settings where there are limited testing supplies. If the result of the pooled test is negative, then none of the individuals are likely to have an active COVID-19 infection. Otherwise, if the pooled test is positive, the individuals are re-tested by taking a new sample from everyone to determine which samples are positive. Screening programs are essential for testing individuals regularly (e.g., daily or weekly) to identify COVID-19 positive cases and catch the outbreaks early on [7]. Surveillance testing can be used to estimate the disease burden on the population (e.g., in a school or organization) to help officials understand and evaluate the effectiveness of their mitigation efforts [11].

## 3. Reporting Guidelines for Device Manufacturers and Testing Facilities

The FDA and CDC has set reporting guidelines for the manufactures of COVID-19 test kits and facilities (e.g., testing sites, clinics, and laboratories) where testing is conducted. The device manufacturers are expected to report performance parameters of their tests, such as accuracy (sensitivity, specificity), positive and negative predictive values, and likelihood ratios estimated from statistical software packages (e.g., Stata v.14 and Clinical Calculator 1) [6]. Here, a false negative test result is more worrisome than a false positive test result. A false negative COVID-19 test result has the risks of delayed medical and supportive treatment for the infected person while giving a false sense of security [14]. False negative results may result in skipping to monitor the close contacts of the infected person for disease symptoms, which can lead to increased transmission risks. Besides the performance parameters, the manufacturers are also expected to report other details of their testing protocol, such as the sample type, intended setting and population groups, standard operating procedure, instructions for use, associated equipment and consumables to perform the test, turnaround time, sample disposal procedure, training requirements, and potential risks to the user and health personnel [4].

Considering the wide variety of COVID-19 tests, testing laboratories are required to ensure that all test reports are complete, comprehensive, unambiguous, and with improved semantic interoperability [2,15]. For this, laboratory testing data across healthcare systems are harmonized and normalized according to the LOINC and SNOMED codes as defined by the CDC’s Laboratory In Vitro Diagnostics (LIVD) Test Code Mapping for SARS-CoV-2 Tests [2]. Testing laboratories are required to report information on the nature of the test ordered, test result, test result date, specimen identifier, patient identifier and demographics, device identifier, ordering provider details, and performing facility details. In addition, hospitals and clinics are required to fill Ask at Order Entry questions for COVID-19 testing: Is this the patient’s first test? Is the patient employed in a healthcare setting? Is the patient symptomatic? Date of onset of symptoms? Was the patient hospitalized because of COVID-19? Was the patient admitted to the intensive care unit (ICU)? Does the patient reside in a congregate care setting? Is the patient pregnant? [2,16].

To efficiently regulate the safety of different COVID-19 tests, the FDA’s Medical Device Reporting (MDR) guidelines requires the device manufacturers and testing facilities to report any device that caused or contributed to death or serious injury within 30 days of becoming aware of the event [17]. Any concerns regarding the adverse events, suspected adverse events, or issues with the test results or test performance can be reported to MedWatch, which is the FDA’s Safety Information and Adverse Event Reporting Program [18].

## 4. Molecular Diagnostic Tests for SARS-CoV-2 Using Respiratory Specimens

**Figure 2:**
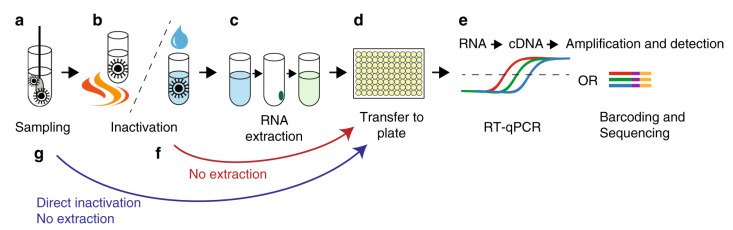
Steps for the reverse transcriptase PCR (RT-PCR) COVID-19 testing. (**a**) The sample is collected and put in a transport medium. (**b**) The virus is inactivated by detergents or by heating. (**c**) Viral RNA is extracted. (**d**,**e**) The contents are transferred to a 96 well plate for cDNA synthesis and detection by RT-qPCR or sample barcoding and high-throughput DNA sequencing. (**f**,**g**) In alternate method, the RNA extraction step can be omitted through direct inactivation of the virus by heating or direct lysis by detergents. The figure is reproduced and adapted under the Creative Commons license from Ioanna Smyrlaki et al., Nature Communications, Springer Nature (2020) [19].

Molecular diagnostic tests detect the presence of SARS-CoV-2 RNA, typically from nasopharyngeal samples, and are considered the gold standard for COVID-19 diagnostics [11]. The nasopharyngeal sample is mailed to a CLIA (Clinical Laboratory Improvement Amendments)-certified laboratory to extract the viral RNA for the RT-PCR (reverse transcription polymerase chain reaction) test as shown in Figure 2. The results are received within 3 to 5 business days. Today, the large suppliers of instruments and analyzers, such as Roche Diagnostics, ThermoFisher Scientific, Abbott, Qiagen and Quest Diagnostics, are expanding their production and testing capacity to perform an increasing number of laboratory tests daily. The list of molecular diagnostic test kits with at-home sample collection and RT-PCR laboratory testing for respiratory specimens granted FDA EUA is shown in Table 1.

**Figure 3:**
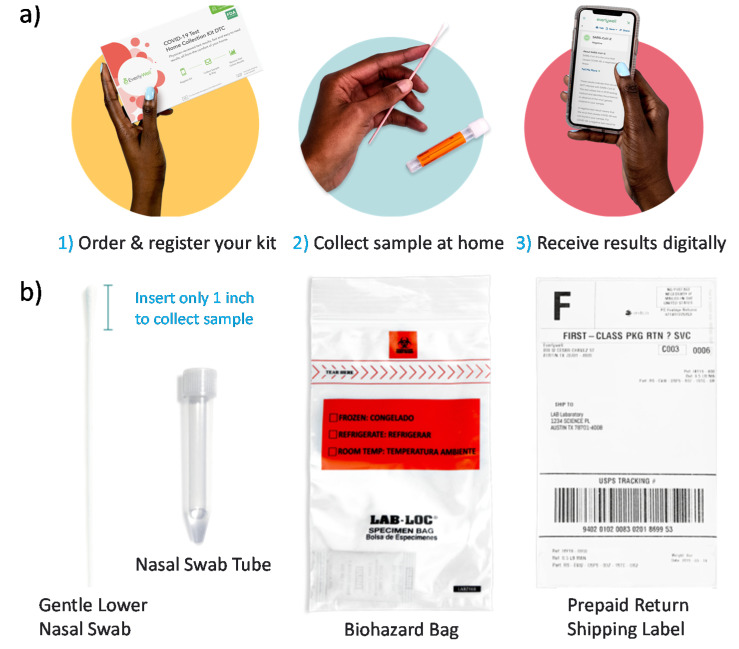
Everywell’s COVID-19 Test Home Collection Kit DTC (direct-to-consumer). (**a**) The procedure involves ordering the test kit, completing a health intake survey, registering the unique kit ID, self-collecting a nasal swab, shipping overnight to a CLIA-certified laboratory, and obtaining RT-PCR results within 24 to 48 h of the lab receiving the sample. A telehealth consult is available to follow up on the test results. (**b**) The Everywell test kit consists of a gentle lower nasal swab, nasal swab tube, biohazard bag, and prepaid shipping label. Images are reproduced and adapted with permission from Everywell [20].

Commercialized molecular diagnostic products differ in the steps of procuring the test kit, conducting the testing procedure, and displaying the results. To highlight these differences, we describe the example products from Everywell, Quest Diagnostics, Curative-Korva, and Lucira Health. The Everlywell COVID-19 Test Home Collection Kit is available over-the-counter through online retailers and local pharmacy stores (Figure 3) [20]. The user takes the health intake survey and registers the unique kit ID at the company website. The sample is self-collected using a nasal swab and shipped to its CLIA-certified laboratory for RT-PCR tests. The results are obtained digitally within 24 to 48 h after receiving the sample. The QuestDirect COVID-19 Active Infection Test from Quest Diagnostics provides an anterior nare (or nasal) swab collection kit for patients who are 18 years or older [21]. The patient has to be pre-qualified by their healthcare provider based on the symptoms, exposure levels, and risk factors. The Quest self-collection kit is delivered to the patient to collect the upper respiratory sample, which is then shipped back in a prepaid envelope. Quest Diagnostics also offers pharmacy drive-thru options for the observed collection of nasal samples.

**Figure 4:**
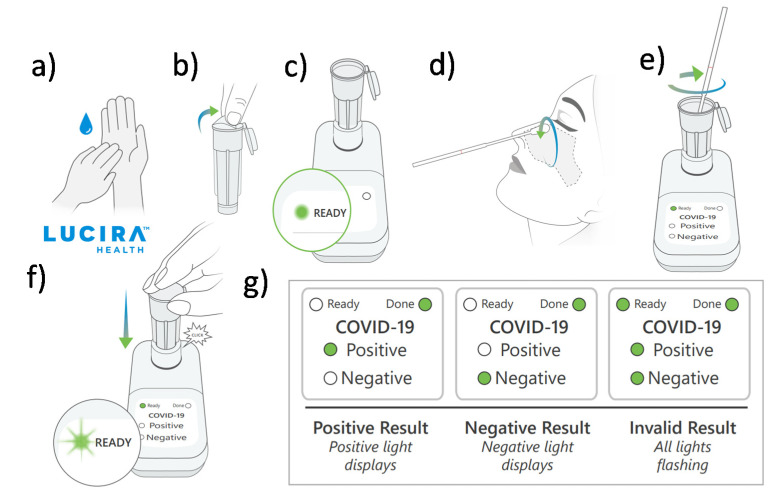
Instructions for using the Lucira COVID-19 All-in-One Test Kit. This involves at-home nasal swab collection followed by RT-LAMP reaction to detect the SARS-CoV-2 genetic material. The steps for use are as follows: (**a**) Wash and dry hands. (**b**) Remove seal from Sample Vial. (**c**) Set the sample vial in the test unit. (**d**) Swab both nostrils. (**e**) Insert swab into the sample vial. (**f**) Snap the cap closed and press the vial into the test unit. (**g**) Wait 30 min and obtain the result. Images are reproduced and adapted with permission from Lucira Health [25].

**Figure 5:**
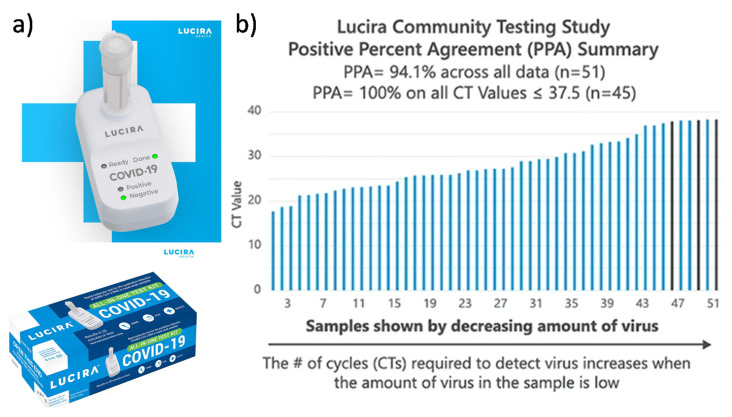
Lucira’s COVID-19 All-In-One Test Kit product and community testing data. (**a**) The single-use, over-the-counter test kit intended for the molecular detection of SARS-CoV-2 RNA from nasal swab samples. (**b**) In a Community Testing Study, the Lucira test was compared with high sensitivity Hologic Panther Fusion SARS-CoV-2 test, and achieved a 94% positive percent agreement (PPA) and a 98% negative percent agreement (NPA). Images are reproduced and adapted with permission from Lucira Health [25].

The Curative-Korva SARS-CoV-2 Assay is intended for prescription use where the respiratory specimen is collected by a trained healthcare provider [22]. The sample is put in Zymo DNA/RNA Shield and shipped to the KorvaLabs Inc. laboratory (San Dimas, CA) to perform the RT-PCR test [22]. Curative has set up over 10,000 COVID-19 testing and health service sites across the U.S. (e.g., drive-thru, kiosks, and mobile sites) and processes over one million tests every week at its three high-capacity labs [23]. The City of Riverside, for example, has collaborated with Curative Inc. to provide free oral fluid COVID-19 tests to all its residents, whether or not they are experiencing COVID-19 symptoms [24]. The Lucira COVID-19 All-In-One Test Kit is authorized for prescription home use with self-collected nasal swabs (in individuals aged 14 and older) and provider-collected nasal swabs (in individuals aged 13 and older) [25]. Instructions for using the Lucira test kit is shown in Figure 4. A community-based testing study found that the Lucira test had a 94.1% positive percent agreement (PPA) and a 98% negative percent agreement (NPA) with a FDA authorized SARS-CoV-2 test as depicted in Figure 5 [25].

## 5. Saliva-Based Molecular Diagnostic Tests for SARS-CoV-2

**Figure 6:**
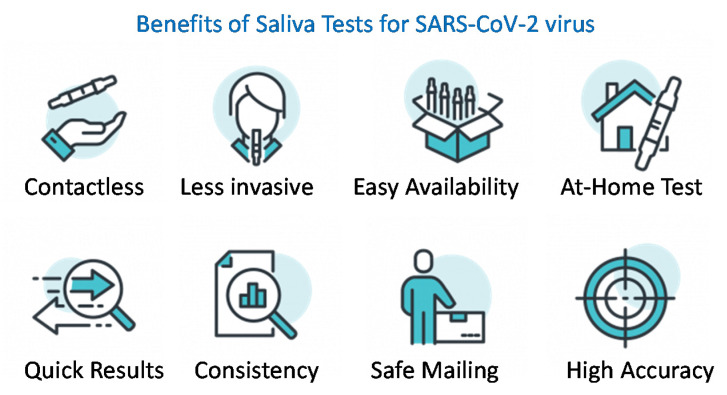
Benefits of using saliva specimens for SARS-CoV-2 detection compared to respiratory specimens. Images are reproduced and adapted with permission from IBX Infinity Biologix [26].

There are benefits of using saliva samples over nasal or throat swabs for the detection of SARS-CoV-2 (Figure 6) [26]. Saliva collection is contactless and poses a reduced risk to the healthcare worker. Saliva collection is less invasive than swab insertions and contributes to lower consumption of personal protective equipment (PPE) and nasal swabs. Saliva testing can be made easily accessible as at-home tests for patients at increased risks, sick patients, or patients in quarantine. However, the SARS-CoV-2 RNA in saliva specimens is only detectable during the acute phase of infection [27,28]. Positive test results from saliva specimens would suggest that SARS-CoV-2 RNA is present but may not rule out infections from bacteria or other viruses. Negative results from saliva specimens may not rule out SARS-CoV-2 infection and must be confirmed with alternate specimens and testing methods [26].

**Figure 7:**
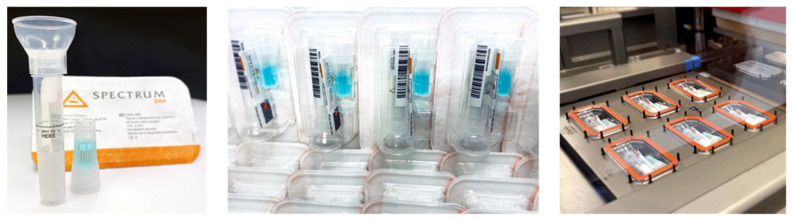
Spectrum Solution’s SDNA collection kit for molecular diagnostics of saliva. The self-collection device is capable of live viral neutralization at room temperature to mitigate risk exposure and sample preservation to identify viral RNA with only 200 copies/mL. Images are reproduced and adapted with permission from Spectrum Solution [27].

At-home saliva collection kits from Spectrum Solution and Infinity BiologiX (IBX) are often used for collecting, maintaining, and preserving saliva samples [26]. The Spectrum Solution’s SDNA-1000 Saliva Collection Device is intended to collect the saliva specimen in a plastic 6 mL tube with the aid of a funnel (Figure 7) [27]. Then, the stabilizing solution, housed in its fluid chamber, is mixed with the saliva specimen. The SDNA-1000 preservation agent inactivates the virus and renders the saliva specimen non-infectious, while preserving the stability of viral RNA for over 14 days at room temperature.

**Figure 8:**
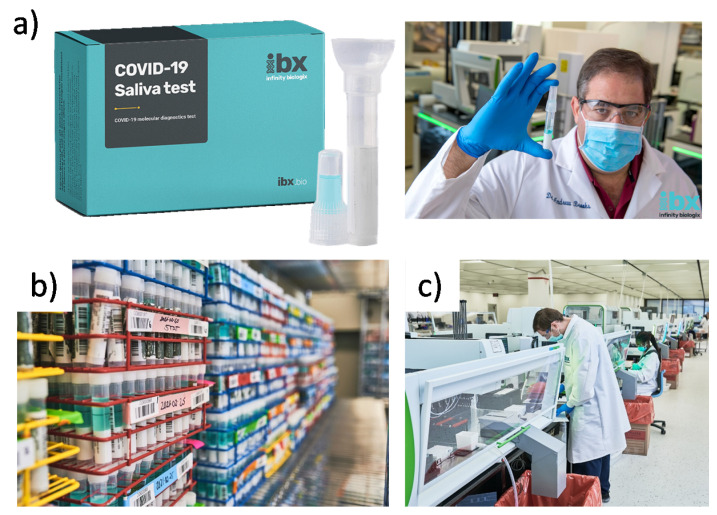
Demonstration of the manufacturing and testing facilities for laboratory processing, data storage, sample preservation, and analytical processes at IBX Infinity Biologix. (**a**) IBX Saliva Collection Kit. (**b**) A repository for cryo-preserving and storing a vast number of biological samples and consumables. (**c**) Automated instrumentation for sample purification, precision liquid handling, viral nucleic acid extraction and testing, Next Generation Sequencing, and analytical quality control. Images are reproduced and adapted with permission from IBX Infinity Biologix [26].

In April 2020, Infinity BiologiX (IBX, formerly called the Rutgers RUCDR Infinite Biologics) received the first FDA Emergency Use Authorization for its saliva tests to detect the SARS-CoV-2 using RT-PCR test [26]. The company received a revised EUA for at-home saliva sample collection using its IBX Saliva Collection Kit (Figure 8) or the Spectrum Solution’s SDNA-1000 Saliva Collection Device [29]. The saliva samples are shipped to an IBX laboratory for storage, purification of genetic material, PCR amplification of extracted RNA, and quality control checks. IBX acts as a service provider for organizations that have large volume testing needs where the subsequent processing of saliva samples is conducted at IBX laboratories (Figure 8). IBX does not process individual tests, but has partnered with service providers such as VaultHealth, 1health.io, ixlayer, and US Drug Test Centers.

A number of companies are providing at-home saliva collection kits followed by RT-PCR tests at high complexity laboratories as shown in Table 2. As an example, the DxTerity SARS-CoV-2 RT-PCR Test is a real-time RT-PCR test for the detection of SARS-CoV-2 RNA from saliva specimens [30]. The saliva specimens are collected in a Spectrum Solution’s SDNA-1000 Saliva Collection Device (by a healthcare provider or self-administered). The saliva specimens are shipped to its CLIA-certified DxTerity Diagnostics Laboratory (California, USA) to perform the RT-PCR tests, and the results are obtained on a secure platform within 24 to 72 h. The DxTerity COVID-19 Saliva at-Home Collection Kit can be purchased for individual tests at Amazon with prepaid shipping of saliva specimens and laboratory testing [28]. The DxTerity SafeWorkDx is intended for large scale screening of the workforce at worksite or from home.

## 6. Rapid Antigen Tests for SARS-CoV-2

Rapid antigen tests look for specific proteins on the surface of the virus from saliva samples or mid-turbinate and nasal swabs (which is more acceptable to patients than nasopharyngeal swabs). These tests are intended to diagnose symptomatic patients within the first five days of symptoms (when there is significant viral load) [13]. Rapid antigen tests are suited for at-home or point-of-care testing as they are relatively inexpensive and provide results within 10 to 15 min [7]. These tests are suited in settings where timely access to RT-PCR tests is difficult or when immediate patient care decisions must be made. The short turnaround time of rapid antigen tests is critical for the timely detection of new SARS-CoV-2 variants and better containment as it is difficult to scale up RT-PCR testing in high complexity laboratories beyond a certain number of daily tests [11]. However, the accuracy of rapid antigen tests (i.e., sensitivity and cross-reactivity with other coronaviruses) is lower compared to the gold standard RT-PCR tests [11]. Rapid antigen tests for at-home SARS-CoV-2 testing are listed in Table 3.

Commercialized rapid antigen tests differ in their test accuracy, acceptability for public testing, and level of collaboration with organizations. We highlight these differences with the products from the following manufacturers: Ellume, Innova, Everlywell and Access Bio. Ellume has developed a fully at-home diagnostic self-test kit for COVID-19 [31]. The Ellume COVID-19 Home Test involves self-collecting a mid-turbinate nasal swab and plugging it into an analyzer. The analyzed data is transmitted to a smartphone application to display and interpret the results. The test takes 15 min to generate a result and is suitable for individuals 2 years of age and older. In individuals with COVID-19 symptoms, the Ellume test correctly identified 96% of positive samples and 100% of negative samples. In individuals without COVID-19 symptoms, the Ellume test correctly identified 91% of positive samples and 96% of negative samples. The FDA issued an Emergency Use Authorization to the Ellume technology as the first over-the-counter COVID-19 diagnostic test that can be conducted completely at home [32]. The FDA allows the test kit to be sold in drug stores for non-prescription use. In December 2020, the National Institutes of Health (NIH) awarded a $30 million grant for delivering 20 million Ellume COVID-19 Home Tests to the United States [33]. In February 2021, the U.S. Department of Health and Human Services awarded $230 million dollars (USD) to Ellume to scale up their manufacturing of at-home testing kits to around 19 million kits every month by the end of this year [34]. These funds, given through the US Health Care Enhancement Act (HCEA), will enable Ellume to ship out around 100,000 test kits each month from February to July and construct a new production plant in the US [34,35].

The Innova SARS-CoV-2 Antigen Rapid Qualitative Test uses a swab to collect the specimen from nostrils or throat [36]. The swab is put in a tube with the extraction fluid. A small amount of the extraction fluid is placed in the sample well of the Lateral Flow Device (LFD) testing cartridge to perform colloidal gold immunochromatography for the detection of nucleocapsid antigens from SARS-CoV-2 [37]. The result is obtained within 30 min. A confirmatory PCR test is suggested after a positive LFD test. The Innova rapid lateral flow COVID-19 test was used for Liverpool mass testing pilots in England [38]. A study reviewed 8774 Innova tests conducted across different groups, including COVID-19 patients, healthcare workers, armed forces personnel, and students [38]. The overall sensitivity was 76.8%, which rose to over 95% in participants with high viral loads. The overall specificity was 99.68% with a low false positive rate (i.e., 22 out of 6967 tests).

Everlywell has partnered with Ginkgo Bioworks to bring the CareStart COVID-19 Antigen Test from Access Bio to its enterprise customers [39]. This rapid antigen test consists of a disposable lateral flow device to detect the active SARS-CoV-2 virus within 15 min. The CareStart COVID-19 MDx RT-PCR Test detects RNA of the COVID-19 nucleocapsid gene and RNA-dependent RNA polymerase gene in test specimens. Everlywell has agreement to bring the CareStart COVID-19 Antigen test to qualifying clinics, workplaces, government offices, schools, and health plans. Its digital solution, Everlywell Lens, can be used to view test results.

In contrast to tests based on respiratory specimens, Lumos Diagnostics has released the FebriDx system as a rapid, finger-prick blood test to differentiate between viral and bacterial acute respiratory infections within 10 min [40]. To distinguish viral from bacterial respiratory infections, the FebriDx system measures the levels of C-reactive protein (CRP) and Myxovirus resistance protein A (MxA). The FebriDx system consists of a lateral flow device into which 5 µL blood is drawn by capillary action and the reagents are released by pressing a button. After 10 min, the results are visually inspected in the form of three lines: the CRP line, the MxA line, and the control line. Elevated MxA levels are indicative of viral infections. A clinical study assessed the accuracy of FebriDx in detecting MxA within 251 hospitalized adults suspected of COVID-19 [41]. FebriDx had a sensitivity of 93% (110/118) and a specificity of 86% (112/130) [41]. The study concluded that FebriDx had a high accuracy and could be deployed as a front door triage test.

## 7. COVID-19 Vending Machines and Testing-as-a-Service Platforms

Besides the development of accurate COVID-19 testing platforms, it has been equally important to build a supply chain for the large-scale distribution of test kits and synchronize the processes of sample collection and handling, laboratory testing, and standardized recording of test results. Several companies are providing innovative solutions, such as vending machines dispensing test kits and end-to-end process management, for rapid and convenient COVID-19 testing. Examples of companies in this space are Wellness 4 Humanity, Azova and P23 Labs, Kroger Heath, CVS Health, and 1health.

Wellness 4 Humanity, founded by social entrepreneurs, developed vending machines for COVID-19 test kits in partnership with Swyft Inc. which is a software and technology service company specializing in unattended retail solutions [42]. There are two test kits offered through their vending machines. The first test, TrustPass Rapid Antigen At-Home Test Kit, provides results within 15 min, and has a 97.4% accuracy and 100% specificity. The second test, At-Home Saliva RT-PCR Test, is intended for saliva collection with Spectrum Solution’s SDNA-1000 collection device that inactivates the live virus in the saliva specimen and preserves and stabilizes the viral RNA transcripts during transport for up to 14 days. The collected saliva sample is sent to a partner lab for gold standard RT-PCR test, and the results are obtained in 24 to 48 h with 98.4% accuracy in symptomatic patients. The saliva testing procedure is made scalable where surveillance pool testing can be conducted to monitor and detect COVID-19 infections in a population (e.g., schools, hotels, workplaces, or hotspots) [43]. Wellness 4 Humanity has partnered with corporations, entertainment studios, hotels, and professional sports teams to conduct scaled-up testing of their clients and employees [44].

Kroger Health’s COVID-19 Test Home Collection Kit is intended for the self-collection of anterior nasal swab specimens by individuals (16 years and older) [45]. The steps for sample registration, sample collection, and shipping with return labels are similar to those of Azova. Testing is limited to CLIA-certified laboratories. Through the Kroger Health COVIDCare Testing Program, at-home test kits are available to corporations for testing their employees through its over 2200 pharmacies and 220 clinic locations across the United States. CVS Health has a Return Ready Testing Program for COVID-19 testing for workplaces and school campuses and a complementary symptom monitoring and contact tracing services from Salesforce through its Work.com platform (Accessed on 5 September 2020) [46]. The Azova COVID-19 Saliva PCR test is available at Costco pharmacy locations and has received the FDA Emergency Use Authorization for self-administration at home, office or in pharmacy [47]. Additionally, referred to as P23 Labs TaqPath SARS-CoV-2 Assay, the test is intended for the quantitative detection of SARS-CoV-2 RNA using rRT-PCR test. Initially, an individual completes a FDA-required pre-assessment and registers for a test kit voucher at the Costco website. The P23 At-Home COVID-19 Test Collection Kit is delivered next day to collect specimens as nasal or throat swabs or saliva samples (that are self-collected at-home or in a healthcare facility). Each saliva collection tube has a unique device ID that is registered at the Azova website and serves to link the sample to the test results. After sample collection, the Azova test kit is dropped off at the nearest UPS location, and the results are received on the Azova app within 12 to 48 h after the testing lab receives the sample.

1health offers a Testing-as-a-Service (TaaS) platform to enable end-to-end process management of COVID-19 tests across patients, companies, laboratories, suppliers, telehealth entities, and government [48]. Its turnkey solution provides end-to-end tracking of services from ordering the tests, logistics, and sample collection to testing, reporting, and billing. Moreover, 1health has partnered with technology companies to provide four COVID-19 tests that have FDA Emergency Use Authorization: RT-PCR At-Home Saliva Test (from Infinity BiologiX), At-Home Nasal Swab Test, Antibody Blood Sample Test, and Antigen Nasal Swab Test [48]. Another TaaS company, Innovaccer, has built a cloud healthcare information platform for the real-time tracking of COVID-19 testing results, turnaround time, and compliance [49]. Its COVID-19 specific Management System is designed to help healthcare enterprises to screen a large number of patients, educate them on CDC guidelines, and provide them with telehealth opportunities [49].

## 8. Serology or Antibody Tests for SARS-CoV-2

These tests measure the levels of antibodies (such as immunoglobulins IgG, IgM or total antibodies in blood) to SARS-CoV-2 [11]. Blood samples for antibody tests are collected from finger stick or by a healthcare personnel at a clinic or pharmacy, and the results are obtained within 1 to 3 days. In most individuals, seroconversion may happen within 7 to 11 days after exposure to the virus, and IgG and IgM titers may plateau within 6 days of seroconversion [7,50]. A study of 338 patients recruited from Tongji Hospital (Wuhan, China) found that IgM levels increased in the first week and peaked in the second week [51]. The IgG levels increased after first week and stayed high for a longer period. The study had used a chemiluminescence immunoassay by YHLO Biotech [51]. As such, the serology or antibody tests provide historical information about exposure to the virus, but they cannot diagnose cases of COVID-19 active infection or acute illness. However, antibodies can take days or weeks to develop after an infection and can stay in the bloodstream after several weeks of recovery. The tests also cannot explicitly tell whether neutralizing antibodies are present (i.e., the ones that protect the individual from the virus) or how long this protective immunity is bestowed against the virus. Antibody tests are helpful to determine the prevalence of COVID-19 in individuals, such as to identify those who are at the risk of COVID-19 infection or those who may be potential donors of convalescent plasma for COVID-19 treatment [13]. A list of the serology or antibody test kits granted FDA EUA is shown in Table 4. We describe the differences in commercialized antibody test kits with some examples, namely Cellex, Bio-Rad, DiaSorin, Vibrant America, Lumos Dignostics, Quest Diagnostics, Scanwell Health, and Vanguard Diagnostics.

The Cellex qSARS-CoV-2 IgG/IgM Rapid Test detects IgG and IgM antibodies to SARS-CoV-2 from serum, plasma, or venipuncture whole blood [52]. The specimen is dropped within a sample well with a sample diluent. The lateral flow test device produces a result within 20 min without the need for a reader or lab equipment. The Cellex assay is meant for prescription use only and is suitable as for self-contained, point-of-care (POC) tests [52]. Bio-Rad’s Platelia SARS-CoV-2 Total Ab Assay detects the three antibodies (IgM, IgA, and IgG) for SARS-CoV-2 in a single test [53]. It uses a 1-step ELISA antigen capture with an incubation time of 90 min. DiaSorin’s LIAISON SARS-CoV-2 S1/S2 IgG is a chemiluminescent immunoassay (CLIA) intended for the qualitative detection of IgG antibodies against SARS-CoV-2 in human serum and plasma [54]. Vibrant America received the first FDA Emergency Use Authorization for its test panel to detect COVID-19 antibodies from dried blood spots [55]. The Vibrant COVID-19 Ab Assay is a chemiluminescence immunoassay intended for the qualitative detection and differentiation of IgM and IgG antibodies against 4 COVID-19 antigens in human serum or dried blood spot. The turnaround time is between 24 to 36 h from the receipt of sample. This test cannot be directly ordered by the patients and is not permitted for at-home testing but is available only at the doctor’s office [55].

Lumos Diagnostics has developed COV-ID as a serological test with a IgG/IgM antibody diagnostic kit for the quantitative detection of antibodies to SARS-CoV-2 [40]. Both Lumos products, FebriDx and COV-ID, are CE marked and approved for sale in Europe and specific global markets, but the FebriDx is not commercially available in the United States. Quest Diagnostics has developed a COVID-19 Antibody Test to detect levels of COVID-19 IgG spike proteins from a previous infection or vaccination [56]. This may be useful to evaluate the immune response of the body to the virus or track the level of antibodies over time. Scanwell Health has collaborated with its telehealth partner, Lemonaid Health, to deliver a SARS-CoV-2 serological test manufactured by a Chinese biotechnology company, Innovita [57]. This home-use test kit detects the levels of IgM and IgG antibodies against SARS-CoV-2 in blood. The test is performed in 15 min and the results are shared through a Scanwell app to a healthcare practitioner for consultation.

To combat the ongoing second wave of COVID-19 infections in India, the Defence Research and Development Organization (DRDO) and Vanguard Diagnostics recently developed an indigenous antibody test kit, DIPCOVAN, for the qualitative detection of IgG antibodies in human serum or plasma [58]. This DIPAS-VDx COVID 19 IgG Antibody Microwell ELISA test kit detects the spike and SARS-CoV-2 nucleocapsid proteins. The antibody test kit has been approved by the Indian Council of Medical Research (ICMR) for sero-surveillance and the early detection of COVID-19 with an expected cost of approximately $1 USD per test.

## 9. Diagnostic Tests (IL-6) for the Management of COVID-19 Patients

These tests detect the different biomarkers of inflammation, track the recovery process, and/or help with patient management decisions. For instance, there are in vitro diagnostic tests for the quantitative measurement of interleukin-6 (IL-6) in human serum and plasma as described in Table 5. Because an elevated level of IL-6 is indicative of systemic inflammation, these assays help in identifying severe inflammatory response in patients with confirmed COVID-19 infection. As a note, severe inflammatory response (also referred to as the “cytokine release syndrome” (CRS) or “cytokine storm”) occurs in less than 20% of COVID-19 patients with symptoms such as fever, hypotension, dyspnea, organ dysfunction, and organ failure [59]. An elevated IL-6 is also an inflammatory signature in COVID-19 patients with acute respiratory distress syndrome (ARDS). Besides identifying severe inflammatory response in COVID-19 patients, these immunoassays help in determining the risks of intubation with mechanical ventilations (e.g., using a cutoff of 35 pg/mL in confirmed COVD-19 patients at presentation). On a cautionary note, the IL-6 levels obtained from these immunoassays should not be solely used to make patient management decisions but used in conjunction with other clinical findings, laboratory test results, patient history and epidemiological information [60,61].

Roche Diagnostics’ Elecsys IL-6 assay is an electrochemiluminescence immunoassay (ECLIA) to be used with the cobas e immunoassay analyzers (cobas 8800 and 6800 systems) [59]. The double-antigen complex is magnetically captured on an electrode. The application of voltage on the electrode induces chemiluminescence that is then measured by a photomultiplier. Beckman Coulter’s Access IL-6 assay is a simultaneous, one-step immuno-enzymatic assay to be used with Access immunoassay analyzers such as Access 2, Dxl 600, and Dxl 800 [60]. A sample is added to a reaction vessel containing paramagnetic particles coated with mouse anti-human IL-6, blocking reagent, and alkaline phosphatase conjugate. After incubation, a magnetic field holds the materials bound to the solid phase while the unbound materials are washed away. To the reaction vessel, a chemiluminescent substrate is added, and the generated light is proportional to the IL-6 concentration in the sample. The ADVIA Centaur IL-6 assay by Siemens Healthcare Diagnostics is a chemiluminescent immunoassay and is intended for use with the ADVIA Centaur XP, ADVIA Centaur XPT, and ADVIA Centaur CP systems [62]. The assay utilizes two reagents. The first Lite Reagent consists of monoclonal mouse anti-IL-6 antibody labelled with an ester in buffer. The second Solid Phase reagent consists of anti-IL-6 mouse monoclonal antibody coated paramagnetic microparticles in buffer. The sample is incubated with the two reagents to allow the formation of immune complexes. After incubation and washing, the ADVIA Centaur Acid Reagent and ADVIA Centaur Base Reagent are added to start the chemiluminescent reaction and generate light that is indicative of the amount of IL-6 in the sample.

## 10. Discussion

As more people are getting vaccinated and the public awareness about COVID-19 testing is improving, the conventional test-trace-isolate strategies for SARS-CoV-2 may eventually be replaced by at-home, low-cost, self-testing based on personal preferences [63]. This requires making COVID-19 testing resources easily accessible, affordable, scalable, quicker, and convenient for the general population [14,19]. At present, it is paramount to ramp up population-scale testing in low and middle-income countries by building a sustainable supply chain logistics [12,23]. In this regard, the WHO’s Access to COVID-19 Tools (ACT) Accelerator Diagnostics Partnership is “focused on bringing to market 2–3 high-quality rapid tests, training 10,000 healthcare professionals across 50 countries and establishing testing for 500 million people in Low and Middle-Income countries by mid-2021” [64].

There are certain limitations of available COVID-19 test kits. Firstly, we need to address the high prices of COVID-19 testing while incorporating more functions and data insights [4,7,8]. Most commercial tests cost over $100 (USD) which is very expensive for the masses. One way to reduce the price of laboratory tests could be to develop simpler protocols for RT-PCR testing, for example, through circumventing the RNA extraction step and performing RT-PCR directly on heat-inactivated samples as shown in Figure 2 [19]. Secondly, existing tests are not able to determine whether an individual has an infectious virus (i.e., the virus that is capable of invading cells and reproducing to produce an infection) or measure the degree of infectiousness from the virus. The infectious virus may be present from 2 to 3 days before symptoms develop and up to 12 days after symptoms develop. Thirdly, current diagnostic assays are qualitative, and it would be informative to track the temporal profiles of viral loads during the course of an infection (e.g., asymptomatic or mildly symptomatic cases where viral levels may be subtle) [7,65]. There are unanswered questions on how long the antibodies persist after COVID-19 infection and whether the antibodies confer protective immunity against the virus. Fourthly, most of the available COVID-19 test kits have not been rigorously tested and validated as compared to other medical tests that have been granted full approval from the FDA. For example, antibody testing should not solely be used to conclude a person is immune to the disease because of cross-reactivity and false positives, and other clinical information should be considered as part of a comprehensive testing plan in consultation with the physician. Lastly, it would be worthwhile to integrate the COVID-19 testing with epidemiologic or seroprevalence studies to help public authorities better characterize the COVID-19 spread or immunity within a community. For example, the Seattle Flu Study, initiated by the University of Washington, is providing free at-home testing for COVID-19 in the Greater Seattle area to track the spread of respiratory illnesses and whether everyday devices, such as the Apple Watch, can predict the COVID-19 illness [66].

Clinical studies are underway for the comparative evaluation and validation of commercial rapid antigen tests [7]. Limiting the number of false negatives in rapid antigen tests is very important, especially in persons displaying no symptoms [67]. Several international studies have confirmed the acceptable performance of rapid antigen tests compared to the gold standard RT-PCR tests [65,68]. A recent large-scale study compared the sensitivity of five antigen tests with RT-PCR tests for the detection of SARS-CoV-2 in nasopharyngeal swabs of 1141 patients [65]. The five antigen test kits used in their stud67y were the Ecotest COVID-19 Antigen Rapid Test (Assure Tech, Hangzhou, China), SARS-CoV-2 Antigen Rapid Test Kit (JoysBio Biotechnology, Tianjin, China), Immupass VivaDiag SARS-CoV-2 Ag Rapid Test (VivaChek Biotech, Hangzhou, China), Standard Q COVID-19 Ag (SD Biosensor, Chungcheongbuk-do, Republic of Korea ), and ND COVID-19 Ag test (NDFOS, Seoul, South Korea). An interesting outcome of this study was that the false negatives indicated by the antigen tests generally had very low levels of viable virus presence in the samples. The study suggested that most asymptomatic or mildly symptomatic patients who are missed by antigen tests (but are COVID-19 positive according to RT-PCR) are probably not infectious [65]. Another study compared the performance of a rapid SARS-CoV-2 antigen detection test, Standard Q COVID-19 Ag kit (SD Biosensor, Seoul, South Korea), with the real-time RT-PCR test, Allplex 2019-nCoV Assay (Seegene, Seoul, South Korea) for detection of SARS-CoV-2 in 454 respiratory specimens (mainly nasopharyngeal and throat swabs) [68]. The study found that the rapid antigen assay, Standard Q COVID-19 Ag kit, had comparable accuracy (sensitivity and specificity) with the Allplex 2019-nCoV RT-PCR assay. Another study compared the performance of seven commercial RT-PCR diagnostic test kits identified from the FindDx website: Altona Diagnostics (Hamburg, Germany), BGI (Shenzhen, Guangdong, China), CerTest Biotec (Zaragoza, Spain), KH Medical (Gyeonggi-do, Republic of Korea), PrimerDesign (Southampton, United Kingdom), R-Biopharm AG (Darmstadt, Germany), and Seegene (Seoul, South Korea) [69]. This study found that the PCR efficiency in the selected RT-PCR diagnostic test kits was satisfactory (~96%) and the LOD95 was within a six-fold range (3.8 to 23 copies per mL). Future large-scale studies from international, scientific entities could convince everyone whether rapid antigen tests are a reasonable alternative to RT-PCR [65].

As new variants of the SARS-CoV-2 emerge, there is a pressing need to periodically track the genomic sequence of the new variants and evaluate the clinical and analytical performance of COVID-19 test kits in detecting the strains [7,9]. This entails conducting large-scale clinical trials which requires significant resources and volunteers [10]. Within the United States, we are aware of two randomized control trials that are underway to compare the efficacy of COVID-19 tests [70,71]. The Rapid Onsite COVID-19 Detection study at University of Wisconsin, Madison is aimed at enrolling community participants (5 years and older) to test a rapid saliva-based assay to detect high levels of SARS-CoV-2 RNA consistent with live virus shedding [70]. Each test is completed in one hour in their mobile laboratories and without the need for any specialized equipment. The CATCh COVID-19 study at the Texas Cardiac Arrhythmia Research Foundation is aimed at enrolling 100 participants to compare the effectiveness of rapid tests in detecting antibodies and viral RNA from blood, nasopharyngeal swab, and sputum specimens of the same patient [71]. At present, understanding the limitations of different commercialized technologies for COVID-19 testing and diagnostics is important, such as the inability of available tests to detect the specific variant of SARS-CoV-2 (e.g., Delta variant) [67,72,73].

**Figure 9:**
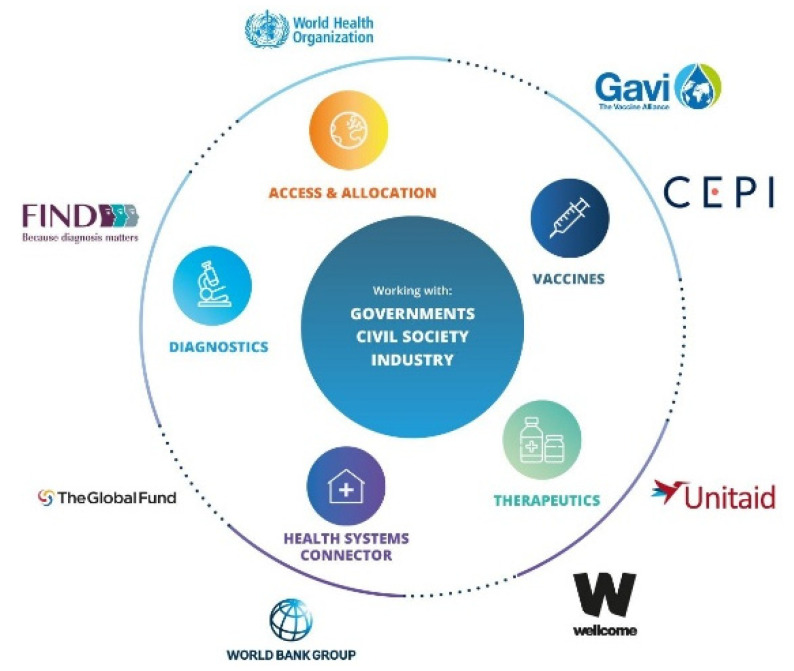
The Foundation for Innovative New Diagnostics (FIND) has partnered with several leading global organizations for the rapid development, scale-up, and universal access to COVID-19 diagnostics, vaccines, and therapeutics. The figure is reproduced under the Creative Commons license from the World Health Organization (WHO) publication [64].

There are certain limitations of our work. In this paper, we primarily covered the COVID-19 assays and test kits that have received Emergency Use Authorization from the FDA [6]. There are other databases of COVID-19 testing platforms with valuable information for the interested readers, such as the websites of Foundation for Innovative New Diagnostics (FIND), the European Commission’s JRC COVID-19 In Vitro Diagnostic Devices and Test Methods Database, and the WHO’s Access to COVID-19 Tools (ACT) Accelerator [4]. The global partnerships for COVID-19 diagnostics, therapeutics, access and allocation, supply chain, and vaccines are shown in Figure 9. Moreover, our perspective on the different tests is based on the public information reported in FDA EUA documents and manufacturers’ websites, which may not reflect the current testing landscape or sample size used for analytical and clinical validation. Lastly, we were unable to establish communication with some manufacturers to inquire about their test kits which could have further improved the scope of this review.

## 11. Conclusions

Different agencies at global and national levels have played a critical role in accelerating the technology development, clinical validation, emergency use authorization, and deployment of IVD tests against the novel coronavirus. While RT-PCR test is considered the gold standard for COVID-19 testing, there is a reasonable case for rapid antigen assays as a low-cost, at-home, self-testing alternative. As more people are getting vaccinated or exposed to SARS-CoV-2, serology or antibody tests will continue to be important in determining the levels of antibodies against the SARS-CoV-2 within different population groups. Today, existing technologies for IVD tests need to constantly improve to be effective against emerging and circulating SARS-CoV-2 variants. Future IVD tests could shine light on several key aspects of the COVID-19 infections, including the temporal profiles of viral loads (e.g., in mildly symptomatic or asymptomatic patients) and longevity of protective immunity provided by the antibodies against SARS-CoV-2. The information discussed in this work will continue to evolve over time as we learn more about different SARS-CoV-2 variants across the world—e it the variant of interest, variant of concern, or variant of high consequence. Finally, the cost associated with COVID-19 diagnostics is significantly high for mass-scale testing in developing and under-developed countries. While local governments and global health organizations are willing to bear a majority of the testing costs today, a sustainable COVID-19 testing model needs to be formulated in the near future that balances the cost, convenience, and quality of IVD tests.

## Figures and Tables

**Table 1:** At-Home Molecular Diagnostic (RT-PCR) Tests using Respiratory Specimens and granted FDA Emergency Use Authorization.

Company Name	Diagnostic Product (EUA Date)	Assay Technology	Test Kit Contents
Alimetrix	Alimetrix SARS-CoV-2 RT-PCR Assay (09/30/2020)	RT-PCR, Microarray Hybridization	Screw-capped collection tube containing 1 mL of 0.85% saline, Specimen biohazard bag (zip-lock) with absorbent pad, ID barcode label for collection tube, instructions
binx health	binx health At-Home Nasal Swab COVID-19 Sample Collection Kit (10/20/2020)	RT-PCR, Direct to Consumer, clinical sample collection method, Testing at High Complexity-certified laboratories	Dry, sterile polyester swab in a tube, specimen bag with absorbent pad, a sample identification label, a return envelope, instructions for use
Clinical Enterprise	EmpowerDX COVID-19 Home Collection Kit DTC (10/15/2020)	Direct to Consumer (DTC), Screening	Sample registration, collection and shipping instructions, Fact Sheet for Individuals, anterior nasal swab, collection tube, collection tube label and pre-labeled FedEx return envelope
Bio-Rad Laboratories	Bio-Rad SARS-CoV-2 ddPCR Test (05/01/2020)	Reverese transcriptase droplet digital PCR test, for prescription use only	Supermix, reverse transcriptase (RT), and 300 mM dithiothreitol (DTT) solution
Becton, Dickinson & Company (BD)	BD SARS-CoV-2 Reagents for BD MAX System (04/08/2020)	Real-time RT-PCR, Serial Screening, Moderate Complexity Test	Lyophilized control template beads for the SARS-CoV-2 Nucleocapsid Phosphoprotein gene (N1), SARS-lyophCoV-2 Nucleocapsid Phosphoprotein gene (N2), and RNase P
Ethos Laboratories	SARS-CoV-2 MALDI-TOF Assay, U-Collect At Home Collection kit (08/03/2020)	RT-PCR and MALDI-TOF Mass Spectroscopy	COPAN swab, Viral Transport Media, specimen bag with absorbent sheet, order form, sample collection and shipping instructions, and a pre-labeled UPS shipping bag
Everlywell	Everlywell COVID-19 Test Home Collection Kit (05/15/2020)	Direct to Consumer, Testing at High Complexity-certified laboratories	Swab and Tube, kit ID stickers, biohazard bag with absorbent sheet, alcohol prep pad, fast sheet, return label, instructions for use
GetMyDNA	GetMyDNA COVID-19 Test Home Collection Kit (03/09/2021)	Direct to Consumer, Screening	GetMyDNA box, biohazard bag, FedEx UN3373 overpack, fact sheet for Individuals, instructions for use
Kroger	Kroger Health COVID-19 Test Home Collection Kit (06/30/2020)	Direct to Consumer, Testing at High Complexity certified laboratories	Fact sheet, patient ID label, alcohol prep pad, biohazard bag, absorbent sheet, nasal swab, transport medium, instruction sheet, return shipping label
LabCorp	COVID-19 RT-PCR Test (03/16/2020)	Real-time RT-PCR, Home Collection, Pooling, Screening	Labcorp At Home COVID-19 Test Home Collection Kit (shipping box, return envelope, specimen biohazard bag, nasal swab, tube)
LetsGetChecked	LetsGetChecked Coronavirus (COVID-19) Test (05/28/2020)	Direct to Consumer, Transcription-Mediated Amplification	Nasal swab, transport tube with media, shipping box, return envelope, instructions, specimen collection materials, biohazard bag with absorbent material, fact sheet
Lucira Health	Lucira COVID-19 All-In-One Test Kit (11/17/2020)	Real-time RT-PCR, Home Collection, RT-LAMP technology	Nasal swab, sample vial, test unit with electronic readout, batteries, disposable bag, quicj reference instructions
Quest Diagnostics	Quest Diagnostics HA SARS-CoV-2 Assay (07/15/2020)	Transcription-Mediated Amplification, chemiluminescent	Instructions for sample collection and shipping, swab, specimen transport tube, Zip-lock bag (biohazard symbol) and desiccant, shipping box, pre-printed label
RapidRona	RapidRona Self-Collection Kit (11/23/2020)	Transcription-Mediated Amplification	Packaged sterile swab, sterile collection tube, transport medium (saline), shipping materials, barcode labels for identification, and instructions for use
ResearchDx, DBA Pacific Diagnostics	PacificDx Covid-19 (12/11/2020)	Transcription-Mediated Amplification, chemiluminescent	Lysis buffer, Applied Biosystems QuantStudio Dx, Proteinase K, RT-qPCR instrument, negative control and positive control
**Specimen Types:** anterior nasal swabs, nasopharyngeal swabs, oropharyngeal swabs, mid-turbinate swabs, nasopharyngeal wash/aspirate or nasal aspirate, and bronchoalveolar lavage fluid			

**Table 2:** At-Home Molecular Diagnostic (RT-PCR) Tests using Saliva Specimens and granted FDA Emergency Use Authorization.

Company Name	Diagnostic Product (EUA Date)	Assay Technology	Specimen Types	Test Kit Contents
Ambry Genetics Laboratory	Ambry COVID-19 RT-PCR Test (01/22/2021)	Real-time RT-PCR, Home Collection, TaqPath COVID-19 Combo Kit, High Complexity Lab Testing certified	Saliva specimen self-collected at home by individuals 18 years and older and deposited within a test tube for processing	Saliva tube label, DNA Genotek OM-505 saliva collection device, bio-specimen bag, absorbent pad, shipping return label, instructions for use
Bio-Rad Laboratories	Bio-Rad SARS-CoV-2 ddPCR Test (05/01/2020)	Reverese transcriptase droplet digital polymerase chain reaction test, for prescription professional use only	nasopharyngeal, oropharyngeal, anterior nasal and mid-turbinate swabs, nasal aspirates, bronchoalveolar lavage fluid	Supermix, reverse transcriptase (RT), and 300 mM dithiothreitol (DTT) solution
Clinical Reference Laboratory (CRL)	CRL Rapid Response (07/30/2020)	Real-time RT-PCR, Home Collection, prescription use only, High Complexity Lab Testing certified	Nasopharyngeal swab, saliva specimen sample	DNA Genotek OM-505 device, saliva collection tube, stabilizing liquid, sealed tube cap, instructions
DxTerity Diagnostics	DxTerity SARS-CoV-2 RT-PCR CE Test (08/28/2020)	RT-PCR, Home Collection, limited to patients with symptoms of COVID-19, High Complexity Lab Testing certified	Nasopharyngeal swab, saliva specimen sample	Spectrum Solutions SDNA-1000 Saliva Collection Device (under healthcare provision), instructions and shipping materials, test tube/cap
Fluidigm Corporation	Advanta Dx SARS-CoV-2 Assay (08/25/2020)	Real-time RT-PCR, Saliva, Home Collection, Online Order, High Complexity Lab Testing certified	Saliva specimen (self collected), 18+ years	Collection tube, device ID label, biohazard bag, instructions for registering and use, absorbent sheet
Gravity Diagnostics	Gravity Diagnostics SARS-CoV-2 RT-PCR Assay (11/23/2020)	Direct to Consumer (DTC), Real-time RT-PCR, Home Collection, Screening, Testing in high complexity certified labs	Anterior nasal, mid-turbinate, oropharyngeal and nasopharyngeal swabs	Spectrum Solutions SDNA-1000 Saliva Collection Device (used under Healthcare Provision), nasal swab, alcohol prep pad, biohazard bag, absorbent sheet
Infinity BiologiX	Infinity BiologiX TaqPath SARS-CoV-2 Assay (4/10/2020)	Real-time RT-PCR, Home Collection, performed in labs certified for high complexity testing	Throat swab (oropharyngeal), nasopharyngeal, mid turbinate nasal, anterior nasal swab, bronchoalveolar lavage fluid, saliva	SDNA-1000 Collection Device, sample collection instructions, shipping materials
P23 Labs	P23 Labs TaqPath SARS-CoV-2 Assay (05/21/2020)	Real-Time RT-PCR, Home Collection, Testing at high-complexity certified labs	Nasopharyngeal swab and saliva, supervised or unsupervised by a healthcare provider	OM-505 Collection Kit (collection funnel/tube, tube cap, collection instructions), biohazard bag, absorbent sheet, U-line box, labeled return overpack
Phosphorus Diagnostics	Phosphorus COVID-19 RT-qPCR Test (06/04/2020)	Real-time RT-PCR, Home Collection, Testing in high complexity certified labs	Oropharyngeal, nasopharyngeal, mid-turbinate, and anterior nasal swabs, nasal aspirates, bronchoalveolar lavage fluid	Kit box, biohazard bag, absorbent pouch, DNA Genotek OGD-510 collection device, test requisition form, instructions for collection
Southern California Permanente Medical Group	Kaiser Permanente High Throughput SARS-CoV-2 Assay (4/19/2021)	Real-Time RT-PCR, Home Collection, 18 years and older, Testing in high-complexity certified labs	Saliva specimens self-collected at home unsupervised using Kaiser Permanente Saliva Home Collection Kit	SDNA-1000 Saliva Collection Device, biohazard pouch, absorbent material, and instructions for use
Wren Laboratories	Wren Laboratories COVID-19 PCR Test (08/03/2020)	Real-time RT-PCR, Home Collection, Testing in high complexity certified labs, symptomatic COVID-19 patients	Nasopharyngeal, oropharyngeal swabs, mid-turbinate nasal, anterior nasal swabs, nasal aspirates, bronchoalveolar lavage fluid, saliva	2 mL uncapped saliva collection tube, funnel mouthpiece, biohazard bag, sealed cap, absorbent pouch, instructions for use
Yale School of Public Health	SalivaDirect At-Home Collection Kit (4/9/2021)	Home Collection Kit, Screening, intended use for patients with and without COVID-19 symptoms	Viral RNA saliva specimens collected using SalivaDirect Unsupervised Collection Kit	SalivaDirect Kit contains options of short straw, funnel, pipette test, and bulb transfer pipette for testing; plastic tube, biohazard bag, alcohol wipe

**Table 3:** Rapid Antigen Diagnostic Tests granted FDA Emergency Use Authorization.

Company Name	Diagnostic Product (EUA Date)	Assay Technology
Abbott Diagnostics Scarborough	(a) BinaxNOW COVID-19 Antigen Self Test (03/31/2021) (b) BinaxNOW COVID-19 Ag Card 2 Home Test (03/31/2021) (c) BinaxNOW COVID-19 Ag 2 Card (03/31/2021)	(a) Lateral Flow, Visual Read, Over the Counter (OTC) Home Testing, Serial Screening (b) Lateral Flow, Visual Read, Over the Counter (OTC) Home Testing, Tele-Proctor Supervised, Serial Screening (c) Lateral Flow, Visual Read, Non-Prescription testing, Serial Screening
Access Bio	CareStart COVID-19 Antigen test (10/08/2020)	Lateral Flow, Visual Read, Serial Screening
Becton, Dickinson and Company	(a) BD Veritor System for Rapid Detection of SARS-CoV-2 (07/02/2020) (b) BD Veritor System for Rapid Detection of SARS-CoV-2 & Flu A+B (03/24/2021)	(a) Chromatographic Digital Immunoassay, Instrument Read, Serial Screening (b) Chromatographic Digital Immunoassay, Instrument Read, Multi-analyte
Celltrion	(a) Sampinute COVID-19 Antigen MIA (10/23/2020) (b) Celltrion DiaTrust COVID-19 Ag Rapid Test (04/16/2021)	(a) Magnetic Force-assisted Electrochemical Sandwich Immunoassay (b) Lateral Flow, Visual Read, Serial Screening
DiaSorin	LIAISON SARS-CoV-2 Ag (03/26/2021)	Chemiluminescence Immunoassay
Ellume Limited	Ellume COVID-19 Home Test (12/15/2020)	Lateral Flow, Fluorescence, Instrument Read, Over the Counter (OTC) Home Testing, Screening
InBios International	SCoV-2 Ag Detect Rapid Test (05/06/2021)	Lateral Flow immunoassay, Visual Read, Serial Screening
LumiraDx	LumiraDx SARS-CoV-2 Ag Test (08/18/2020)	Microfluidic Immunofluorescence Assay, Instrument Read
Luminostics	Clip COVID Rapid Antigen Test (12/07/2020)	Lateral flow immunoluminescent assay, instrument read
Ortho Clinical Diagnostics	VITROS Immunodiagnostic Products SARS-CoV-2 Antigen Reagent Pack and Antigen Calibrator (01/11/2021)	Chemiluminescence Immunoassay, Instrument Read
Princeton BioMeditech	Status COVID-19/Flu A & B (02/04/2021)	Lateral Flow, Visual Read, Multi-analyte, Differential Diagnosis of SARS-CoV-2, Influenza Type A and Type B Antigen
Qorvo Biotechnologies	Omnia SARS-CoV-2 Antigen Test (04/13/2021)	Bulk Acoustic Wave (BAW) Biosensor, Instrument Read
Quanterix Corporation	Simoa SARS-CoV-2 N Protein Antigen Test (01/05/2021)	Paramagnetic Microbead-based Immunoassay
Quidel Corporation	(a) Sofia SARS Antigen Fluorescent Immunoassay (05/08/2020) (b) Sofia 2 Flu + SARS Antigen Fluorescent Immunoassay (10/02/2020) (c) QuickVue SARS Antigen Test (12/18/2020) (d) QuickVue At-Home COVID-19 Test (03/01/2021) (e) QuickVue At-Home OTC COVID-19 Test (03/31/2021)	(a) Lateral Flow, Fluorescence, Instrument Read, Serial Screening (b) Lateral Flow, Fluorescence, Instrument Read, Multi-Analyte (c) Lateral Flow, Visual Read (d) Lateral Flow, Visual Read, Prescription Home Testing (e) Lateral Flow, Visual Read, Over the Counter (OTC) Home Testing, Serial Screening
**Specimen Types:** nasopharyngeal swab or anterior nasal swab (self-collected or observed); **Approved FDA EUA Use:** identification of SARS-CoV-2 nucleocapsid protein antigen		

**Table 4:** Serology or Antibody Tests granted FDA Emergency Use Authorization.

Company Name	Diagnostic Product (EUA Date)	Assay Technology
Abbott Laboratories	SARS-CoV-2 IgG assay (04/26/2020)	Chemiluminescent microparticle immunoassay, IgG
Azure Biotech	Assure COVID-19 IgG/IgM Rapid Test Device (09/23/2020)	Lateral flow chromatographic immunoassay, IgG and IgM
Beckman Coulter	Access SARS-CoV-2 IgG assay (06/26/2020)	paramagnetic particle, chemiluminescent two-step immunoassay, IgG
Bio-Rad Laboratories	Platelia SARS-CoV-2 Total Ab assay (04/29/2020)	Total Antibody, Enzyme-linked immunosorbent assay
Cellex	qSARS-CoV-2 IgG/IgM Rapid Test (04/01/2020)	Lateral flow assay, IgG and IgM
DiaSorin	LIAISON SARS-CoV-2 S1/S2 IgG (04/24/2020); LIAISON SARS-CoV-2 IgM (09/29/2020)	Chemiluminescent microparticle immunoassay, IgG and IgM
Emory Medical Laboratories	SARS-CoV-2 RBD IgG test (06/15/2020)	Enzyme-linked immunosorbent assay, IgG
Hangzhou Biotest Biotech	RightSign COVID-19 IgG/IgM Rapid Test Cassette (06/04/2020)	IgG and IgM, Lateral Flow, fingerstick whole blood
Healgen Scientific	COVID-19 IgG/IgM Rapid Test Cassette (Whole Blood/Serum/Plasma) (05/29/2020)	IgG and IgM, Lateral Flow
InBios International	SCoV-2 Detect IgM ELISA (06/30/2020)	IgM, Enzyme-linked immunosorbent assay
Mount Sinai Laboratory	COVID-19 ELISA IgG Antibody Test (04/15/2020)	IgG, Enzyme-linked immunosorbent assay
Ortho Clinical Diagnostics	VITROS Immunodiagnostic Products Anti-SARS-CoV-2 Total Reagent Pack (04/14/2020)	Total Antibody, Chemiluminescent microparticle immunoassay
Roche Diagnostics	Elecsys Anti-SARS-CoV-2 (05/02/2020)	Total Antibody, Electrochemiluminescence immunoassay
Siemens Healthcare Diagnostics Inc.	Dimension Vista SARS-CoV-2 Total antibody assay (COV2T) (06/08/2020)	Total Antibody, Chemiluminescent microparticle immunoassay
Thermo Fisher Scientific	OmniPATH COVID-19 Total Antibody ELISA Test (10/02/2020)	Total Antibodys(including IgM, IgA and IgG to SARS-CoV-2), ELISA
Vibrant America Clinical Labs	Vibrant COVID-19 Ab Assay (06/04/2020)	IgG and IgM, Chemiluminescent microparticle immunoassay
Wadsworth Center, New York	New York SARS-CoV Microsphere Immunoassay for Antibody Detection (04/30/2020)	Total Antibody, Fluorescent microsphere Immunoassay
**Specimen Types:** human serum, plasma, fingerstick whole blood or venous whole blood; **Approved FDA EUA Use:** human SARS-CoV-2 IgM (immunoglobulin-M), IgG (immunoglobulin-G), and total antibodies		

**Table 5:** Tests for the management of COVID-19 patients (IL-6 assays) granted FDA Emergency Use Authorization.

Company Name	Diagnostic Product (EUA Date)	Materials (Required or Suggested)	Analyzing Systems	Assay Technology Measuring Interval
Siemens Healthcare Diagnostics	ADVIA Centaur IL-6 assay (18 December 2020)	ADVIA Centaur Ancillary Probe Wash, Centaur Wash, IL-6 Quality Control	ADVIA Centaur XP, XPT, CP systems	One-step direct chemiluminescent IL-6 immunoassay; 3.0–5500.0 pg/mL
Beckman Coulter	Access IL-6 assay (1 October2020)	Access IL-6 Calibrators, Access Sample Diluent A, Access Substrate, Access 2 UniCel DxC 600i, IL-6 Quality Control	Access 2, Dxl 600, Dxl 800 systems	One-step immunoenzymatic (“sandwich”) IL-6 assay; 2–1500 pg/mL
Roche Diagnostics	Elecsys IL-6 assay (2 June 2020)	IL-6 CalSet, PreciControl Multimarker, Diluent MultiAssay, ISE Cleaning Solution/Elecsys SysClean	Cobas 8800, 6800 systems	Electrochemiluminescence IL-6 immunoassay; 1.5–5000 pg/mL
**Specimen Types:** human serum or plasma; **Storage conditions:** 2–8 °C for 28 days; **Approved FDA EUA Uses:** (i) Measure IL-6 levels in COVID-19 patients; (ii) Identify severe inflammatory response from elevated IL-6; (iii) Determine the risk of intubation with mechanical ventilation				

## Data Availability

Data is contained within the article.
